# Quantification of Peptides in Food Hydrolysate from *Vicia faba*

**DOI:** 10.3390/foods14071180

**Published:** 2025-03-28

**Authors:** Jean Manguy, Georgios I. Papoutsidakis, Ben Doyle, Sanja Trajkovic

**Affiliations:** Nuritas Limited, Joshua Dawson House, 19B Dawson Street, Dublin 2, D02 RY95 Dublin, Ireland; manguy.jean@nuritas.com (J.M.); papoutsidakis.georgios@nuritas.com (G.I.P.);

**Keywords:** peptidomics, food hydrolysate, peptide, absolute quantification, mass spectrometry

## Abstract

The hydrolysis of raw food sources by commercially available food-grade enzymes releases thousands of peptides. The full characterization of bioactive hydrolysates requires robust methods to identify and quantify key peptides in these food sources. For this purpose, the absolute quantification of specific peptides, part of a complex peptide network, is necessary. Protein quantification with synthetic tryptic peptides as internal standards is a well-known approach, yet the quantification of non-tryptic peptides contained in food hydrolysates is still largely unaddressed. Similarly, data analyses focus on proteomic applications, thus adding challenges to the study of specific peptides of interest. This paper presents an in-sample calibration curve methodology for the identification of three non-tryptic peptides present in a *Vicia faba* food hydrolysate (PeptiStrong™) using heavy synthetic peptides as both calibrants and internal standards.

## 1. Introduction

Plant-based peptides, typically consisting of 2–30 amino acids, are found either naturally in plant sources or released from parent proteins during hydrolysis [1,2]. These peptides exhibit diverse biological functions, including inhibiting insect feeding, contributing to plant defence mechanisms, and regulating cell division and reproduction processes [3].

The bioactivity of plant-based peptides is typically inactive until released through fermentation or hydrolysis during food processing. The composition of the resulting food hydrolysate varies based on factors like the raw food source, its origin, growth season, and storage conditions. Hydrolysis occurs in large bioreactors through several steps: protein extraction, solubilization, enzyme addition, hydrolysis, enzyme inactivation, and final biopeptide characterization. Enzymatic hydrolysis, the core process, requires the precise control of environmental conditions such as pH and temperature for optimal results [4,5].

Plant peptides have been associated with a wide range of health benefits, including antioxidant [6,7], anticarcinogenic [8,9], antimicrobial, antihypertensive, immunomodulatory [7,10], and cognitive function improvements [11,12].

The structural integrity of bioactive peptides must be maintained to ensure their bioactivities before reaching their site of action, requiring resistance to degradation by gastrointestinal and serum peptidases. Peptide bioavailability depends on factors like charge, hydrophobicity, and hydrodynamic volume [13]. Challenges in measuring peptide bioavailability include low concentrations in food matrices and serum, along with susceptibility to enzymatic degradation [14]. Gastrointestinal stability is a key factor influencing peptide bioavailability, with more research needed to determine whether peptides are more stable within protein hydrolysates due to peptide interactions [15]. Additionally, food matrix components and intestinal conditions can affect absorption [16,17]. Clinical trials assess biopeptide stability, but alternative in vivo models, like simulated gastrointestinal models and Caco-2 cell lines [18,19], and ex vivo models, such as Ussing chamber [19], are also used. Nevertheless, tissue-based models are the first approach [20].

The absorption of di- and tripeptides through the apical membrane is well documented, but little is known about their transport through the basolateral compartment [19]. Peptides that survive digestion are typically hydrolyzed by serum and endothelial peptidases, making them difficult to detect or quantify [21,22]. Challenges to peptide absorption include diffusion through tight junctions, enterocytes, and enzymatic digestion by cytosolic enzymes [21,23], although some tripeptides were reported to be absorbed intact [24]. Larger peptides face even more difficulties with conflicting findings on their absorption [25]. Finally, the knowledge of peptide plasma concentrations and their kinetic mechanism for the proper assessment of peptide bioactivity in humans is required, where absorption evidence is lacking. The quantification of biopeptides in complex food matrices is necessary, prior to any assessment of stability, bioavailability, and absorption studies. For a hydrolysate to exhibit the same level of bioactivity from batch to batch, or to assure the same quality of hydrolysis, it is important to quantify these key peptides in each batch. The batch-to-batch reproducibility of the hydrolysate is rarely reported [26]. Some of the released peptides are predicted, through artificial intelligence (AI), to have biological activity, which is then confirmed with bioassays [27].

The traditional quantification method for peptides typically involves constructing a calibration curve using a blank matrix spiked with synthetic peptides or their heavy isotope-labelled versions [28]. However, this approach is not ideal for food hydrolysates, where peptides of interest are surrounded by numerous other peptides, proteins, carbohydrates, fats, and small molecules, leading to complex matrix effects. Such effects can affect ion suppression, sample recovery, and signal intensity, ultimately compromising peptide quantification [29].

Peptidomics, which adapts proteomic techniques [28], faces challenges in peptide selection for quantification. Unlike proteomics, where the best tryptic peptide is selected from a set, peptidomics requires identification of the exact peptide [30]. Furthermore, proteomic methods typically target peptides with higher charges (+2 or more), which aids in identification, while food hydrolysate peptides often carry a +1 charge, complicating both identification and quantification [31,32].

While relative quantification has been explored for biomarker discovery [33], the absolute quantification of peptides in plant-based food hydrolysates remains poorly studied. Existing methods, such as 1-point calibration curves and multiple reaction monitoring (MRM), are not suitable for this purpose [34,35]. However, reversed internal calibration curves and targeted parallel reaction monitoring (PRM) methods show promise for accurate peptide quantification in food hydrolysates [36,37]. To that end, the aim of this project is to adapt methods from proteomics and develop an absolute quantification method for peptides by using an in-sample calibration curve on a Q Exactive instrument (ThermoFisher Scientific, Inc., Canoga Park, CA, USA).

## 2. Materials and Methods

We developed a method to quantify three peptides within a hydrolysate (PeptiStrong™) with the precise addition of C^13^ labelled, synthetic versions of these same peptides using previously characterized proprietary *V. faba* PeptiStrong™ [27]. The latter was manufactured from fava bean food material (*V. faba*) and supplied from our ingredient laboratory. This hydrolysate was previously characterized in Cal et al., (2020) and in Corrochano et al., (2021) [27,38].

**Figure 1:**
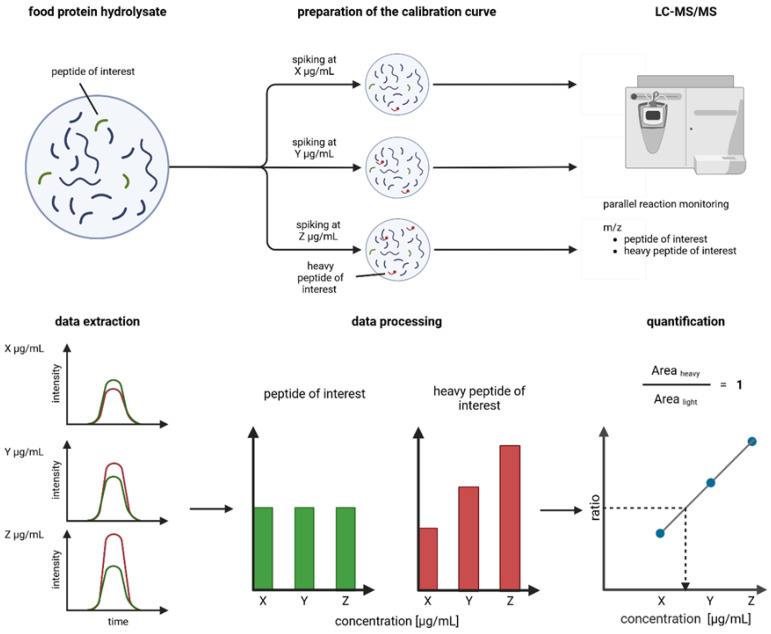
Outline of the preparation and analysis of internal calibration curves to quantify a peptide of interest in a food protein hydrolysate. Colors red and green match between panels.

Figure 1 outlines the main experimental steps of this work to perform the absolute quantification of peptides of interest in PeptiStrong™. Details about materials and methods are available in the Supplementary Information.

We selected three peptides for quantification based on their bioactivity and sequence: HLPSYSPSP, HLPSYSPSPQ, and TIKIPAGT. The bioactivity of HLPSYSPSPQ and TIKIPAGT for protein synthesis increase and TNF-alpha reduction was previously predicted and validated [27]. HLPSYSPSP and HLPSYSPSPQ were added together due to their sequence similarity. All three peptides were previously identified in LC-MS/MS DDA runs. There was no further step to include or exclude peptides. Heavy-labelled peptides were synthesized (GenScript, Piscataway, NJ, USA) for each peptide of interest (Table 1). One residue per peptide was selected to be a heavy amino acid. Different residues for HLPSYSPSP and HLPSYSPSPQ were chosen to avoid the overlap of MS2 fragments.

### 2.1. Sample Preparation

Hydrolysate powder PeptiStrong™ (0.2 g) was dissolved in water (4 mL), and the solution was vortexed for 30 s. The solution was centrifuged at 4000 rpm for 20 min at 4 °C and filtered through a 0.2 µm filter. The protein/peptide content of the filtrate was determined using a Bicinchoninic acid assay (BCA) (see Supplementary Materials).

Volumes from the stock solution of the hydrolysate were normalized to 1000 µg of protein/peptide and added to low-binding Eppendorf tubes. The samples were evaporated to dryness in a rotary vacuum evaporator (Concentrator Plus, Eppendorf, UK). Dried samples were resolubilized in 1000 μL of LC-MS grade solution of 0.1% *w*/*w* formic acid in water. Heavy-labelled peptides, TIK[+8]IPAGT, HLP[+6]SYSPSPQ, and HLPSYSPSP[+6], were solubilized in LC-MS grade water at 1 mg/mL according to the net weight declared by the manufacturer.

### 2.2. Preparation of Broad and Refined Internal Calibration Curves

**Figure 2:**
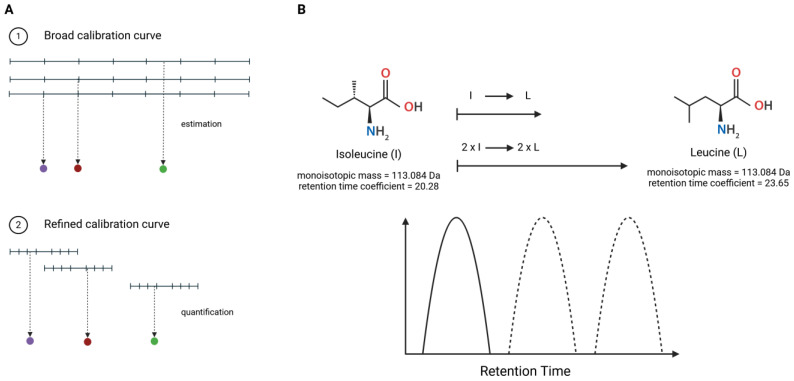
(**A**) Building peptide-specific internal calibration curves (refined) from generic calibration curves (broad); (**B**) Illustration of the observable retention time difference between isoleucine and leucine [39]. Colors match between panels.

Two sets of calibration curves were prepared for this work. The first set of broad calibration curves for each peptide of interest, was prepared using the same concentrations of each heavy peptidoforms, covering a broad range of values. The second set of calibration curves was a refinement of the broad calibration curves. Following the peptide quantification with the broad calibration curves, a refined calibration curve was constructed for each peptide, using the assessed concentrations of the light peptidoform as the mid-point (Figure 2A). The hydrolysate sample (250 µg) was spiked with various concentrations of heavy peptides to a final volume of 1 mL for the construction of both types of calibration curves. Namely, 1 mL solution of 0.1% *v*/*v* formic acid contained 250 µg of PeptiStrong™ hydrolysate and the corresponding concentrations of heavy peptidoforms outlined in Supplemental Tables S1 and S2.

Following the spiking of the hydrolysate, the solutions (1 mL) were processed through solid phase extraction (Supplementary Information) and evaporated to dryness. The samples were resuspended in 21 µL of Pierce Retention Time Calibration Mix (PRTC) in 0.1% formic acid in water, centrifuged for 5 min at 15,000 rpm, and transferred to LC-MS vials, for analysis via mass spectrometry.

### 2.3. Parallel Reaction Monitoring Mass Spectrometry Method and Inclusion List

Samples were analyzed in triplicate by nano LC-MS/MS Dionex UltiMate 3000 coupled to a ThermoFisher Q Exactive™ in positive polarity mode. A trapping column was utilized for loading peptides, which were subsequently eluted over a 25 cm analytical column PepMap RSLC C18 with a 70 min gradient at a flow rate of 300 nL/min. The gradient started with 95% mobile phase A (99.9% LC/MS grade water with 0.1% LC/MS grade formic acid) and 5% mobile phase B (99.9% LC/MS grade acetonitrile with 0.1% LC/MS grade formic acid) for 2 min, increased to 20% mobile phase B over 48 min and up to 75% mobile phase B for an additional 20 min. This gradient was held constant for 10 min followed by a 13 min re-equilibration step at a flow rate of 350 nL/min and a 2 min pressure re-equilibration step at a flow rate of 300 nL/min.

The mass spectrometer was operated in full MS and parallel reaction monitoring (PRM) modes. The full MS method was performed in the Orbitrap positive mode at 17,500 FWHM resolution with AGC target of 3 × 10^6^, maximum IT of 50 ms, and scan range from 500 to 2000 *m*/*z*. PRM method was performed at 35,000 FWHM resolution with AGC target of 2 × 10^5^ and maximum IT of 100 ms. Isolation window was 4.0 *m*/*z* with fixed first mass of 120.0 *m*/*z* and normalized collision energy of 28. The inclusion list was built from the selected peptides and their heavy peptidoforms. Each sample was injected 3 times.

### 2.4. Peak Integration

The peak selection and integration in mass spectrometry raw files were conducted with Skyline (MacCoss Lab Software; Version 22.2.0.312) [40,41]. The peptides of interest, previously identified from a data-dependent acquisition (DDA) run using PEAKS (Bioinformatics Solution Inc., Waterloo, ON, Canada; Version 1.5) [42], were used as spectral library. Other b and y ions were added to the Skyline integration to identify fragments that were not found in the DDA run. The peptide sequences were modified to include heavy proline and heavy lysine at their respective positions. Skyline was set to search for heavy isotope label types and to use the light peptide as internal standard. Manual inspection of the peaks was performed to confirm and correct the retention time, peak integration time, and selected transitions for quantification for each peptide. Data were then exported to CSV files from Skyline for downstream analysis.

### 2.5. Peptide Sequence Validation

The selected peptides were identified using open database searches from DDA experiments [43]. All 3 peptides contain leucine or isoleucine residues; thus, the exact sequence of these peptides might be incorrect due to the similar mass of these 2 amino acids. During the previous peak integration step, we screened the retention time shifts between light and heavy peptides. If required, additional PRM runs were performed using synthetic light and heavy peptides in water. The analysis of the extracted ion chromatogram (XIC) of these runs was used to test the hypothesis that the retention time shift observed in the MS runs of the spiked hydrolysate could be explained by the difference in hydrophobicity between amino acids leucine and isoleucine.

### 2.6. Linear Regression and Peptide Quantification

A linear regression fit was applied for each peptide by using the corresponding ratio of heavy and light to the on-column concentration of spiked heavy peptides on the x-axis. The concentration of each peptide of interest was calculated with inverse estimation of a ratio, with 1 representing the equal quantity of heavy and light peptides.

The limit of quantification was determined for the broad calibration curve by performing a segmentation analysis of the linear regression model using the segmented R package [44,45]. The breaking point between the two resulting linear regressions represents the point between the linear response and the noise. Segmentation analysis was not possible for the refined calibration curve. Thus, the limit of quantification was determined by using 10 times the slope of the calibration curve divided by the standard deviation of the peak area ratio at the y-intercept.

## 3. Results

### 3.1. Validation of Peptide Sequence Using Synthetic Heavy Peptides

**Figure 3:**
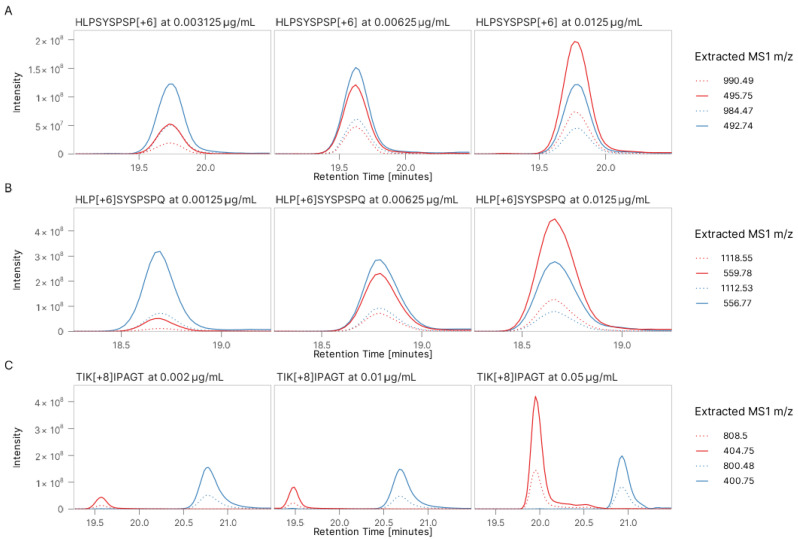
Peptide sequence validation: (**A**) extracted ion chromatogram (XIC) of peptide HLPSYSPSP (blue line) and its spiked heavy-labelled peptide (red line) at three different concentrations (0.003125–0.0125 μg/mL, red line) in food hydrolysate PeptiStrong™; (**B**) XIC of peptide HLPSYSPSPQ (blue line) and its spiked heavy-labelled peptide (red line) at three different concentrations (0.00125–0.0125 μg/mL, red line) in food hydrolysate PeptiStrong™; and (**C**) XIC of peptide TIKIPAGT (blue line) and its spiked heavy-labelled peptide (red line) at three different concentrations (0.002–0.05 μg/mL, red line) in food hydrolysate PeptiStrong™.

Peptide TIKIPAGT/TIKLPAGT from *V. faba* [27] was previously characterized by other DDA experiments. We assumed the peptidoform with isoleucine was the correct sequence. Figure 3 shows the XIC of the corresponding light endogenous peptide and its spiked, heavy-labelled counterpart in hydrolysate PeptiStrong™. Figure 3A,B show the identical retention time between peptides HLPSYSPSP and HLPSYSPSPQ with their spiked, heavy-labelled counterparts, whereas the retention time for peptide TIKIPAGT and its spiked heavy-labelled counterpart does not match in the XIC profiles, although both peptide sequences were confirmed at the MS2 level (Supplemental Figure S2).

**Figure 4:**
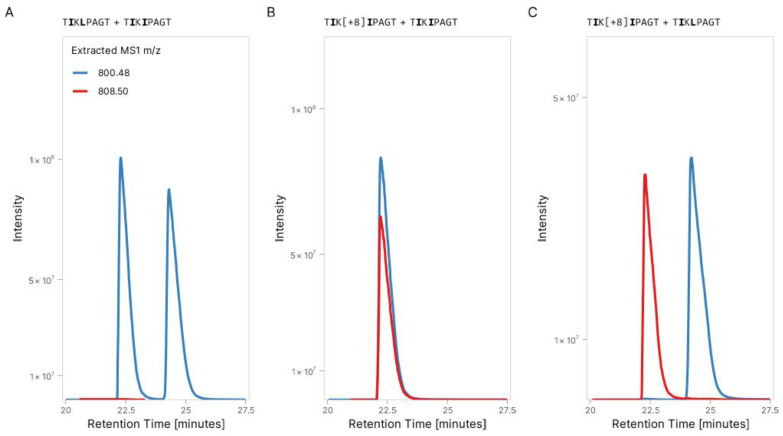
Mass spectrometry resolves the sequence of isoleucine-leucine isoforms using a heavy-labelled peptide: (**A**) XIC (*m*/*z* tolerance = 0.1) analysis for synthetic peptides TIKIPAGT and TIKLPAGT with two different peaks for *m*/*z* 800.48 at different retention times. (**B**) Comparison of XIC for light synthetic TIKIPAGT and heavy-labelled TIKIPAGT peptides shows two peaks for *m*/*z* 800.48 and 808.50, respectively, at the same retention time. (**C**) Comparison of heavy-labelled TIKIPAGT and synthetic TIKLPAGT peptides shows peaks for *m*/*z* 800.48 and 808.50, respectively, at different retention times. The retention times are consistent for each isoform across conditions.

We posited that this difference in retention time is most likely caused by a leucine being present instead of an isoleucine in the sequence of TIKIPAGT due to their different retention time coefficients (Figure 2B). Peptides with the TIKLPAGT or TLKIPAGT sequences would elute approximately at the same time; peptides with the TIKIPAGT sequence would elute before, and peptides with the TLKLPAGT sequence would elute after. To validate these predictions, we performed an experiment using the synthetic peptides of both light and heavy TIKIPAGT peptides with synthetic light TIKLPAGT. Figure 4A–C represent different conditions of an experiment with different combinations of light and heavy peptidoforms in water using the same analytical parameters as the absolute quantification method. Figure 4A shows the difference in retention time between TIKIPAGT and TIKLPAGT. The peak of the endogenous peptide does not match with the peak of the heavy version of peptide TIKIPAGT as it is the isoform of leucine (TIKLPAGT). We identified TIKIPAGT with confidence as the first peak at 22.3 min and TIKLPAGT as the second peak at 24.2 min. Thus, hereafter, we refer to this peptide using the sequence TIKLPAGT.

### 3.2. Estimation of Concentration for Multiple Peptides with Broad Internal Calibration Curves

**Figure 5:**
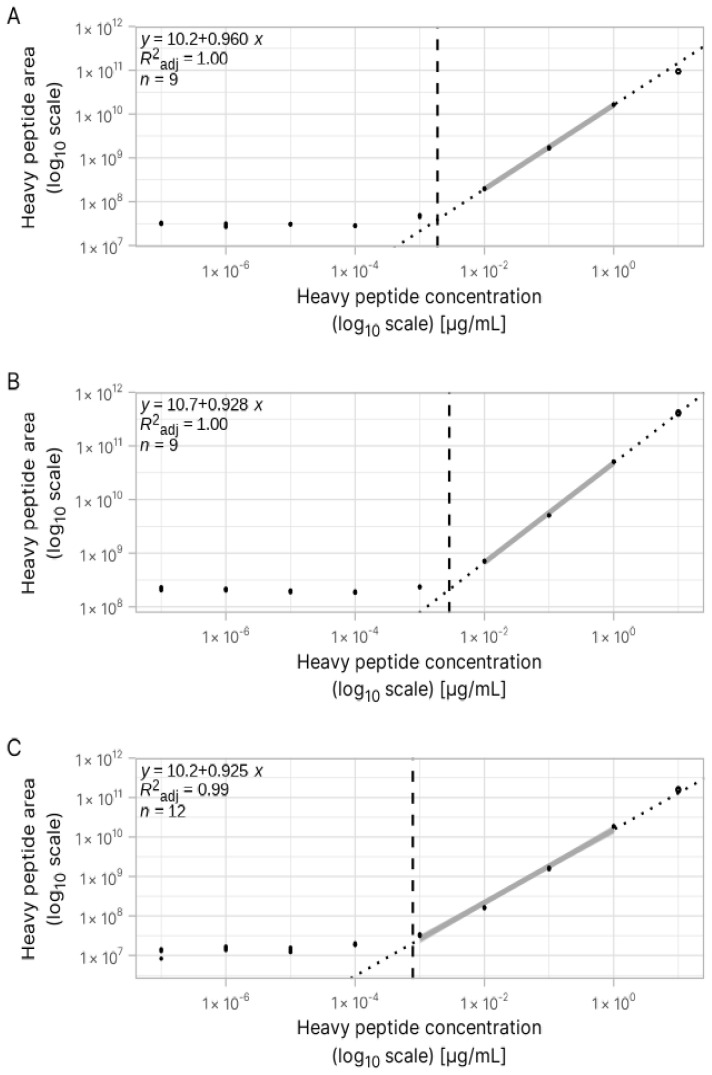
The peak area of the heavy peptide (**A**) HLPSYSPSP[+6], (**B**) HLP[+6]SYSPSPQ, and (**C**) TIK[+8]IPAGT is plotted against its concentration in a PeptiStrong™ hydrolysate matrix. The linear regression is segmented to separate the linear part of the calibration curve versus noise. The vertical dashed line represents this breaking point. The highest concentration is also excluded from the calibration curve due to saturation of the signal observed during the peak integration.

In a reversed, in-sample calibration curve with heavy-labelled peptidoforms, the signal of the quantified (light) peptide is constant, while the signal for the heavy peptidoform varies based on its increasing concentration (Figure 3). Figure 5 shows that the linear relationship between the concentration and signal lies within a concentration range for the heavy-labelled peptide. A wide range of concentrations is used for the construction of a broad calibration curve; thus, many points fall outside the linearity range. We used a segmentation analysis on a linear regression model between the concentration and peak area of the heavy peptide to find the breaking point between noise and the linear part of the calibration curve (Figure 5). The linear curve for heavy-labelled peptide HLPSYSPSP[+6] (Figure 5B) displays the lowest LOQ value, 0.00079 μg/mL, compared to 0.0031 and 0.002 μg/mL for heavy-labelled peptides HLP[+6]SYSPSPQ and TIK[+6]IPAGT, respectively.

### 3.3. Absolute Quantification Using Peptide-Specific, Refined Calibration Curves

The broad set of calibration curves cannot be used to accurately quantify the peptides of interest as there are not enough data points to compose the linear part of the calibration curve. Thus, we constructed a second, more refined set of calibration curves for each peptide of interest using the estimated concentrations from their corresponding broad calibration curves.

**Figure 6:**
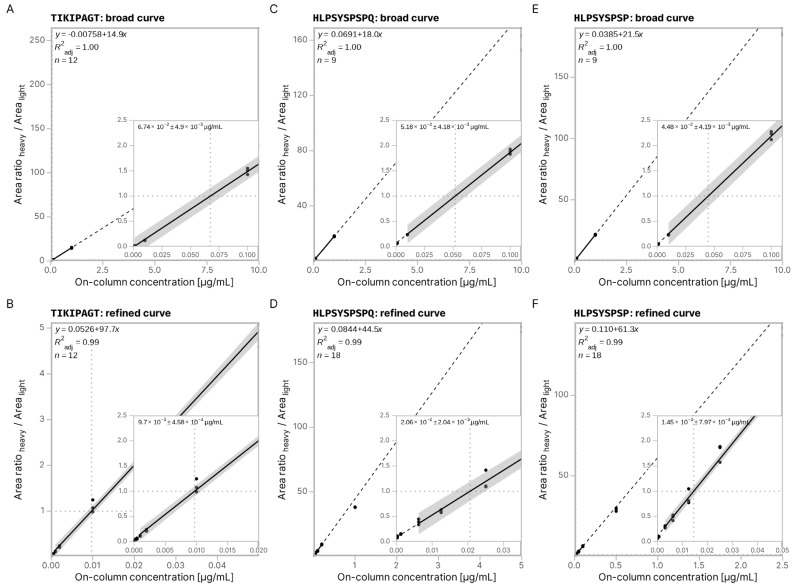
Absolute quantification of peptides using in-sample calibration curves: (**A**,**C**,**E**) Broad calibration curves for peptides TIKLPAGT, HLPSYSPSPQ, and HLPSYSPSP. (**B**,**D**,**F**) Refined calibration curves for peptides TIKLPAGT, HLPSYSPSPQ, and HLPSYSPSP. Each panel shows the linear regression between heavy peptide concentration and the heavy peptide peak area after segmentation between the flat response and the linear response. Points outside of the linear range are manually removed from the calibration curve. The linear regression used for calibration is shown with a solid black line (95% confidence interval). For illustration purposes, the line is continued above and below using a dotted line. The on-column concentration of each peptide is estimated through the ratio of the concentration of the heavy peptide over the corresponding of the endogenous peptide of 1, herein shown with a horizontal dotted line.

Figure 6 shows the higher accuracy of the peptide-specific refined calibration curves compared to the broad calibration. The broad calibration curves of each peptide (Figure 6A,C) show that the estimated concentration values differ by several orders of magnitude from the values calculated with the refined calibration curve. We calculated the following concentrations for the peptides of interest in the tested hydrolysate: TIKLPAGT at 0.01 ± 7 × 10^−4^ µg/mL; HLPSYSPSQ at 0.021 ± 0.002 µg/mL; and HLPSYSPSP at 0.015 ± 8 × 10^−4^ µg/mL.

## 4. Discussion

In protein-focused quantification experiments, peptides can be selected based on previous mass spectrometry data to choose the best surrogates. The methodology presented here aims to quantify a set of previously selected peptides in a food hydrolysate. In this context, as the peptides are the focus, no selection step is possible. In addition, proteolysis with an analytical-grade trypsin enzyme is relatively deterministic. However, peptides from food hydrolysates can be the product of different hydrolysis steps, using one or more different chemicals or proteases that may be operating in non-optimal conditions. A resultant matrix will contain multiple peptides with similar sequences because of the unspecific and/or partial hydrolyses. These peptides might co-elute, and fractionation might release identical ions, causing issues with identification and sensitivity. This matrix is also very likely to contain peptides without any proton-capturing amino acids, exacerbating these issues.

To limit matrix effects and ion suppression effects, we chose to use in-sample calibration curves using synthetic heavy peptidoforms of the peptides of interest. Any matrix effects are applied to both the standards and the peptides quantified. We used an PRM method to target only the light and heavy peptides required to build the calibration curves. We chose this approach as the analyte is measured consistently with PRM, and the limit of detection (LOD) is found when detection is absent, while the lack of identification with the DDA type of acquisition indicates that the LOD was either reached or the analyte was not sampled [46]. DDA experiments that involve an increased proteome coverage suffer from under-sampling problems and lead to the under-representation of low abundance analytes [47]. MRM, on the other hand, monitors multiple product ions from one or more precursor ions, while PRM simultaneously analyzes all fragment ions derived from the precursor ion with high mass accuracy and resolution [48]. PRM offers higher specificity and reproducibility than SRM due to high-resolution and accurate mass analyzers, which minimize signal interference [49]. The sensitivity of PRM and SRM are similar, but the former outperforms DIA (data-independent acquisition) in both sensitivity and specificity for targeted peptides [50,51].

We also built calibration curves using different concentrations for each peptide. Because each peptide has different physicochemical properties and experiences different matrix effects, the possible linear range of the calibration curve is different for each peptide. We decided to build a generic broad calibration curve using the same concentrations for all heavy peptides. The analysis of the results from this first calibration curve informed the construction of a second calibration curve, specific for each peptide.

In this study, we quantified three peptides originating from the same food hydrolysate from *V. faba.* We chose two of them based on their bioactivity, which was previously predicted and validated [27], and the third was chosen based on the sequence similarity to one of the first two peptides. The quantification of these three peptides in a food hydrolysate was successful. We show that we successfully built a robust calibration curve for each peptide. Additional work was performed to prove that the retention time difference between the endogenous and synthetic peptidoforms of the TIKLPAGT peptide was caused by the isoleucine/leucine amino acids. While we successfully built a valid calibration curve for this peptide, the heavy and light peptides were not under the exact same matrix effects and did not experience the same ion suppression effects. Usually, isoleucine and leucine in peptide sequences are treated as the same amino acid by search algorithms. Mass spectrometry alone cannot differentiate between the two. Matching MS spectra from plant-based food hydrolysates to peptide sequences can be made more difficult depending on the quantity and quality of the protein sequences from the plant species that can be used by peptide search engines. Progress is being made to improve de novo peptide identification [52], but such methods may not be able to discriminate between isoleucine and leucine. Additional work is needed to evaluate the impact of peptide sequence errors on the quantification of peptides, that were caused by these amino acids, as presented here.

The limited number of peptides that can be reliably targeted using PRM is a drawback [53]. Further work is needed to define which peptides and how many peptides can be quantified at once with this method. Other more advanced targeted methods are promising and should be investigated in the context of a food hydrolysate [54].

The approach presented here has already been applied to biomedical research. In this study, we show that it can be applied in multiple food hydrolysate research experiments, primarily experiments focused on few peptides of interest. This methodology is resource-intensive and may not be suitable for various routine applications such as quality control or food stability experiments. In addition to the technical limitations discussed above, the increased resources required to quantify additional peptides do not make this methodology a good candidate for experiments aiming to quantify numerous peptides at once in one hydrolysate. However, we show here a robust method to quantify key bioactive peptides from a plant-based food hydrolysate.

The development of precise and standardized quantification methods for peptides in food supplements and functional products is crucial to characterize their positive and measurable bioactive and nutritional effects [55,56]. It is also capable of reducing uncertainty in the prediction of unintended negative effects [57]. Indeed, the absolute quantification of peptides within a plant-based hydrolysate unlocks applications in bioavailability experiments and in vitro bioactivity assays.

## 5. Conclusions

We successfully resolved the retention time shift between isoleucine and leucine peptidoforms to identify the correct sequence of one of our peptides of interest using a spiked heavy-labelled peptide. This sequence difference would not have been resolved with a simple DDA run followed by standard peptide search. We achieved the absolute quantification of multiple peptides in a single hydrolysate using a PRM method targeting peptides of interest and their heavy peptidoforms. The heavy peptidoforms served two purposes: they were utilized both as calibrants to construct a five-point calibration curve and as internal standards to address the losses due to sample preparation and matrix effects. The refined calibration curves did not validate the corresponding broad calibration curves; thus, it is not sufficient to implement one generic set of concentrations in a calibration curve to fit all the experiments for peptide quantification.

## Figures and Tables

**Table 1:** Peptide sequences (using the proforma v2 format) and their molecular weight values used in this work.

Peptide of Interest		Spiked Internal Standard	
Peptide Sequence	Molecular Weight (Da)	Peptide Sequence	Molecular Weight (Da)
HLPSYSPSP	984.4785	HLPSYSPSP[Label:13C(5)15N(1)]	990.4923
HLPSYSPSPQ	1112.5371	HLP[Label:13C(5)15N(1)]SYSPSPQ	1118.5509
TIKIPAGT	800.4876	TI[Label:13C(6)15N(2)]KIPAGT	808.4876

## Data Availability

The original contributions presented in this study are included in the article/Supplementary Materials. Further inquiries can be directed to the corresponding author.

## Supplementary Materials

The following supporting information can be downloaded at https://www.mdpi.com/article/10.3390/foods14071180/s1, Supplementary Materials and Methods: Materials, Heavy Peptides, Bicinchoninic Acid Assay, Solid-Phase Extraction; Figure S1: Selection of precursor ions matching the peptide of interest in noisy spectra; Figure S2: Identification of light and heavy peptides; Table S1: Broad calibration curves for each quantified peptide; Table S2: Refined calibration curves for each quantified peptide.

## Abbreviations

The following abbreviations are used in this manuscript:

PRM	Parallel reaction monitoring
MRM	Multiple reaction monitoring
SRM	Selected reaction monitoring
XIC	Extracted ion chromatogram
DDA	Data-dependent acquisition
DIA	Data-independent acquisition
BCA	Bicinchoninic acid assay
