# Supplementary material for: Quantification of Peptides in Food Hydrolysate from Vicia faba
Source: Foods. 2025 Mar 28;14(7):1180. doi: 10.3390/foods14071180 (PMC11988565; doi:10.3390/foods14071180)
Supplement: Supplementary file 1 [file foods-14-01180-s001.zip › foods-3513869-supplementary.pdf]

Quantification of Peptides in Food Hydrolysate from *Vicia faba*

Supplementary online content

Supplementary Materials and Methods

Materials

The hydrolysate was supplied from the manufacturing facility at Nuritas [1]. The chemical compounds utilized were solutions of bovine serum albumin (P0834, 10 × 1 mL, Protein Standard, 2 mg protein/mL, Albumin, SLCK1200), for the construction of calibration curve for the bicinchoninic acid (BCA) assay, Bicinchoninic acid (B9643-1L-KC, Lot: SLCG8086), copper (II) sulfate (4% w/w, C2284-25 mL-KC, Lot: SLCF1912) from Sigma, water (Optima LC/MS grade, 2.5 L, W6-212) acetonitrile (optima, LC/MS grade, 2.5 L, A955-212, Lot: 2188409), methanol (Optima, LC/MS grade, 2.5 L, A456-212, Lot: 2191283), formic acid solution (optima, LC/MS grade, 50 mL, A117-50, Lot: 185797), syringe filters (PES, 0.2 μm, sterile, Cat. No. 15206869, 33 mm) from Fisher, Pierce™ HeLa Protein Digest Standard (5×20 μg, 88329, Lot: XB343350) and trifluoroacetic acid solution (LC/MS grade, 50 mL, purity ≥ 90, 85183, Lot: 2188409) from Thermo, and extraction cartridges (PRiME HLB, 1cc (30 mg), 186008055, Lot: 015831195A, Oasis) from Waters. Pioneer™ analytical balance (Model: PA223, OHAUS®) with repeatability 0.001 g was used for the preparation and dilution of samples.

Heavy peptides

Synthetic peptides TIK[+8]IPAGT (1.5 mg, purity 96.8%, Lot: U9887HD260-16/PE5082), TIKIPAGT (1.5 mg, purity 98.7%, Lot U575WHF280-5/PE7325), HLPSYSPSP (1.5 mg, 98.5% purity, Lot U9887HD260-43/PE5109), HLPSYSPSP[+6] (1.5 mg, purity 96.7%, Lot U9887HD260-46/PE5112), HLPSYSPSPQ (1 mg 97.3% purity, Lot U784UFA240-1/PE1495), and HLP[+6]SYSPSPQ (1.5 mg, purity 96.8%, Lot: U9887HD260-16/PE5082) were provided by Genscript (NJ, USA).

Bicinchoninic acid assay (BCA)

Bovine serum albumin (BSA) stock solution (2 mg/mL) was prepared by adding 10 mg of BSA protein powder into 5 mL of deionized water. Protein standards were prepared (0-2000 μg/mL) using deionized water. Hydrolysate sample sh_YO75MA was diluted 10-fold or 20-fold in deionized water to ensure that the values lied within the linear

part of the BSA calibration curve. Bicinchoninic acid and copper (II) solutions were mixed in a 50:1 ratio. Protein standards and diluted sh_YO75MA sample (25 μL) were added in triplicates to a 96-well plate. Prepared BCA assay solution (200 μL) was added to the standards and sh_YO75MA samples, and the plate was equilibrated at 37 °C for 30 minutes. The absorbance was then measured in a microplate spectrophotometer reader (CLARIO star, BMG LABTECH) at 562 nm. The protein/peptide concentration of the samples was measured by interpolation of the readings of the samples in the equation of the calibration curve using the CLARIOstar MARS data analysis software. The appropriate volume was added to a new 1.5 mL low-binding Eppendorf tube after normalization to 1000 μg of protein/peptide content, and the samples were evaporated to dryness in a rotary vacuum evaporator.

Solid-Phase Extraction (SPE)

Solid-phase extraction (SPE) took place with an automated liquid handling system (Agilent, UK). The samples were prepared to a concentration of 1 mg/mL and acidified with TFA solution to a final concentration of 0.2% v/v. The extraction HLB cartridges with their pressure caps were added to the cartridge racks, the elution tubes were added to the elution racks, and the acidified samples were added to the sample rack. A sample volume (1 mL) was loaded onto each cartridge, and once this volume went through, the cartridges were washed with 1 mL of 5% methanol in water. The retained peptides were eluted with 2 × 250 mL 70% organic solvent (90:10 acetonitrile and methanol, respectively)/30% deionized water solution. The eluted samples were evaporated to dryness in a rotary vacuum evaporator, were solubilized in 62.5 μL Pierce™ retention time calibration mix (10 μL added in 990 μL of 0.1% v/v TFA solution), centrifuged for 5 minutes at 15000 rpm, and were added to MS for analysis.

Supplemental Figure S1: Selection of precursor ions matching the peptide of interest in noisy spectra.

Supplemental Figure S2: Identification of light and heavy peptides.

Supplemental Table S1: Broad calibration curves for each quantified peptide.

Supplemental Table S2: Refined calibration curves for each quantified peptide.

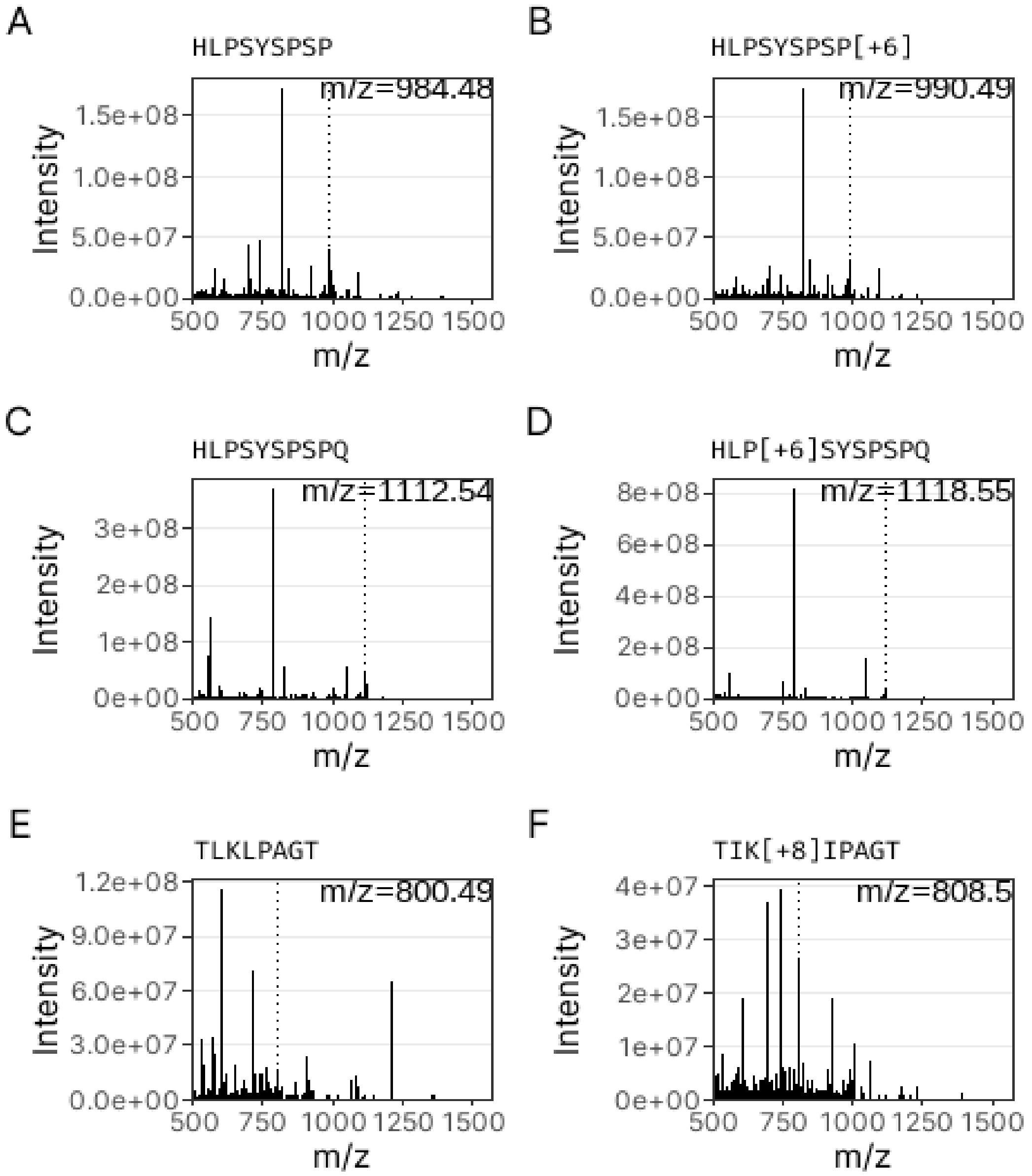

Supplemental Figure S1: Selection of precursor ions matching the peptide of interest in noisy spectra. The PRM targeted method allows for a specific selection of ions that may not be selected for a standard DDA run. The dotted line represents the selected precursor matching for each peptide.

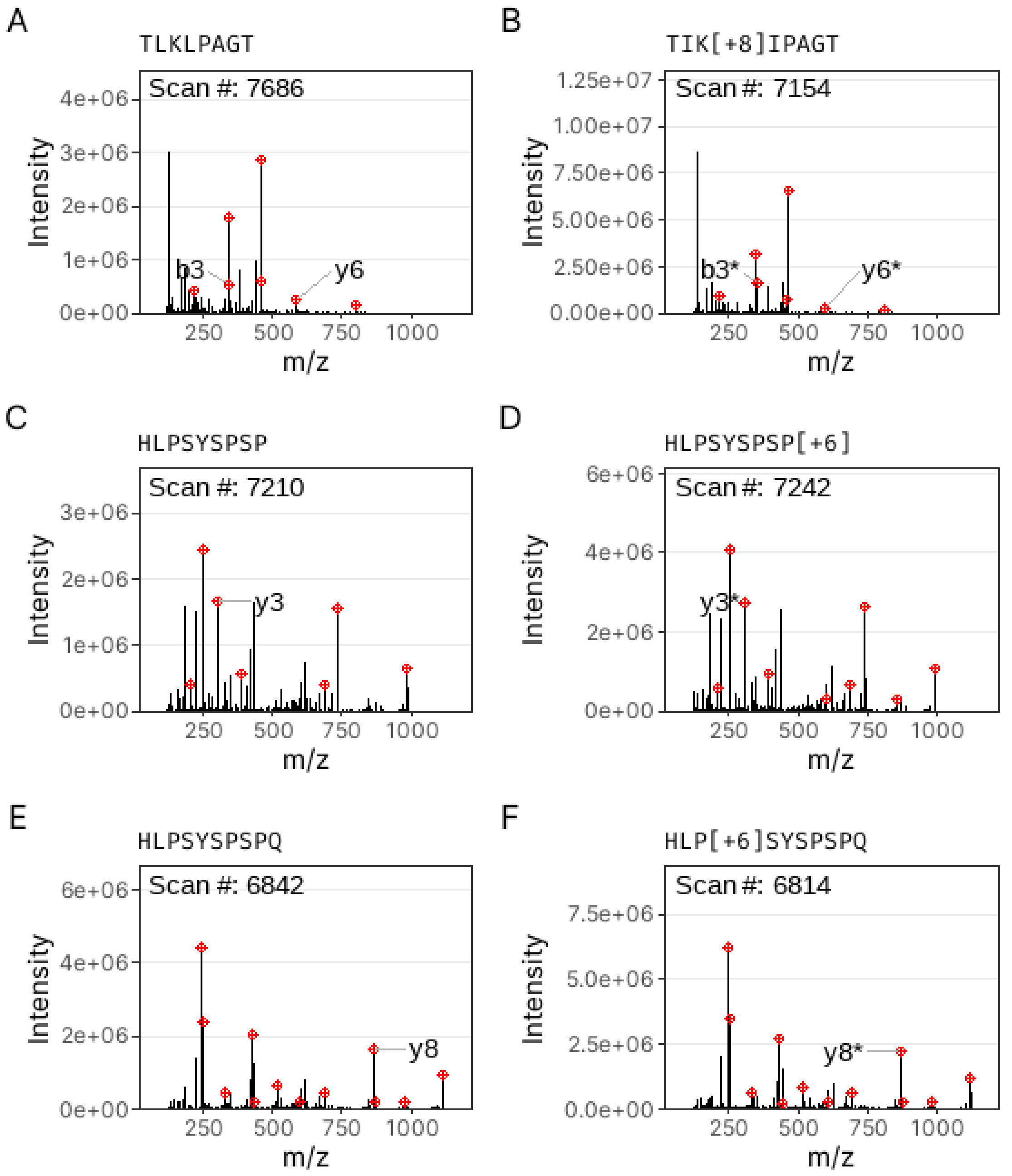

Supplemental Figure S2: Light and heavy peptide identification. The red targets identify the ions above the median intensity in the spectra matching each peptide (m/z tolerance = 0.01). The transitions at the heavy amino acid position are annotated. The asterisk indicates the transition with the heavy amino acid.

Supplemental Table S1. Broad calibration curves constructed from the serial 10x dilution of the heavy peptidoforms of each quantified peptide.

Sample	On-column heavy peptide concentration [μg/mL]		
	TIK[+8]IPAGT	HLP[+6]SYSPSPQ	HLPSYSPSP[+6]
Broad 1	10	10	10
Broad 2	1	1	1
Broad 3	0.1	0.1	0.1
Broad 4	0.01	0.01	0.01
Broad 5	0.001	0.001	0.001
Broad 6	0.0001	0.0001	0.0001
Broad 7	0.00001	0.00001	0.00001
Broad 8	0.000001	0.000001	0.000001
Broad 9	0.0000001	0.0000001	0.0000001

Supplemental Table S2: Refined calibration curves constructed from the results of the broad calibration curves. The fifth sample represents the middle point of these new curves.

Sample	Dilution	On-column heavy peptide concentration [μg/mL]		
		TIK [+8] IPAGT	HLP[+6]SYSPSPQ	HLPSYSPSP[+6]
Refined 1	100 x	0.03494	3.528	1.8045
Refined 2	20 x	0.006988	0.7056	0.3609
Refined 3	4 x	0.0013976	0.14112	0.07218
Refined 4	2 x	0.0006988	0.07056	0.03609
Refined 5	1 x	0.0003494	0.03528	0.018045
Refined 6	½ x	0.0001747	0.01764	0.0090225
Refined 7	¼ x	0.00008735	0.00882	0.00451125
Refined 8	1/8 x	0.000043675	0.00441	0.002255625
Refined 9	1/40 x	0.000008735	0.000882	0.000451125
Refined 10	1/200 x	0.000001747	0.0001764	0.000090225

References

[1] Corrochano A R, Cal R, Kennedy K, Wall A, Murphy N, Trajkovic S, O'Callaghan S, Adelfio A and Khaldi N 2021 Characterising the efficacy and bioavailability of bioactive peptides identified for attenuating muscle atrophy within a Vicia faba-derived functional ingredient *Curr. Res. Food Sci.* **4** 224–32
